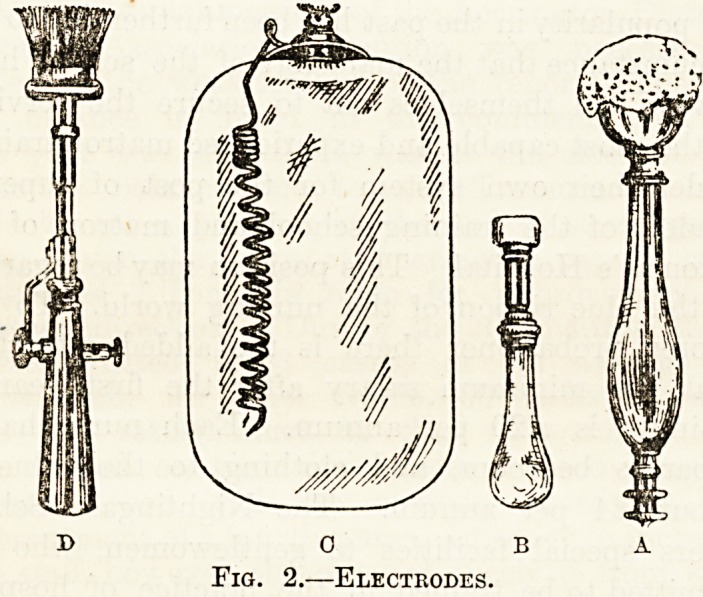# The Hospital. Nursing Section

**Published:** 1905-07-22

**Authors:** 


### The Hospital.

### Nursing Section.

Contributions for this Section of "The Hospital" should be addressed to the Editor, "The Hospital" Nursing Section, 28 & 29 Southampton Street, Strand, London, W.C.

No. 982.—Vol. XXXVIII. SATURDAY, JULY 22, 1905.

### Notes on News from the Nursing World.

### THE NEW RED CROSS SOCIETY.

The council of the new Red Cross Society which was inaugurated at a meeting at Buckingham Palace on Monday by a speech from the Queen in person, includes two nurses, Miss Monk, the matron of King's College Hospital, and Miss Ethel McCaul. They will do their best to support the stirring appeal of her Majesty who invites all the women of the Empire to assist her in carrying on the great scheme which, as she finely put it, is the one and only way in which women can assist "our brave and gallant Army and Navy to perform their arduous duties in time of war."

### RETIREMENT OF MISS THOROLD.

We regret to announce the retirement of Miss G. M. Thorold, lady superintendent of Middlesex Hospital, who, owing to family reasons, has tendered her resignation to the committee. Miss Thorold, who has held the matronship for more than 35 years, was trained at University College Hospital, where she entered as a probationer, owing to her great desire to qualify herself to nurse the sick and needy in a parish in North Devon of which her father was rector. A friend of hers was at that time matron of Middlesex Hospital, and when it became essential to find some one to take her place during her vacation, the matron appealed to Miss Thorold, who accepted the invitation. For three years in succession the same request was made, and thus the committee were enabled to judge of Miss Thorold's powers of administration. So highly did they learn to esteem these that later, when the position of lady superintendent became vacant, it was at once offered to the rector's daughter working quietly in her country seclusion amongst the sick cottagers. In the long period which she has spent in the institution in Mortimer Street, Miss Thorold has seen many changes. A special wing has been added for the treatment of the cancer patients, a beautiful convalescent home has been erected at Clacton-on-Sea, the system of three years' training for nurses has been introduced, and a handsome nurses' home has been built in communication with the hospital. In spite of the enlargement, Miss Thorold still does as she did 30 years ago, when there was so much less to direct and see after, learning to know thoroughly both her nurses and her patients. The latter she never fails to visit individually twice a day, speaking to them many words of comfort and help, except when she is absent in the spring time. Miss Thorold works as hard in taking her pleasure as she does in the exercise of her profession. In 1904 she had already visited the Holy Land, Egypt, Greece, Constantinople, Russia, Spain, and America, and accordingly she travelled into that little-known island, Corsica, and was immensely pleased with her experience. This year she spent her vacation amongst the Pyrenees Mountains, visiting Bagnères de Luchon and coming home by way of Touraine so as to see some of the most interesting chateaux in France. Miss Thorold will remain in office till the alterations at the hospital are completed and will receive back the nurses and patients. When Michaelmas comes she will bid them farewell and will reside once more in the country rectory where she lived as a girl and left to come to London. Miss Thorold has taken an active part in all movements for the benefit of nurses, and was for many years one of the keenest supporters of the Royal British Nurses' Association. In her well-earned leisure she will have the satisfaction of knowing that she has done much to raise the standard of nursing, and has endeared herself to all sorts and conditions of people with whom she has been brought in contact. We are sure that she will not be allowed to sever her prolonged connection with the Middlesex Hospital without some appropriate tribute of the respect and esteem in which she is held being offered to her.

### LONG HOLIDAY FOR THE MIDDLESEX NURSES.

A large placard on the railings of the Middlesex Hospital announces to the public that the building is "closed for repairs," and not only are most of the wards already in the hands of the painters, but the Nurses' Home is comparatively empty. The majority of the sisters and nurses have departed for two months' vacation, and are not expected back till September 7th when they will have ten days to get everything in order to receive the patients, to whom the hospital will be reopened on the 17th. There remain in the institution a few patients who are never, in any circumstances, sent away. These are the 36 women and nine men suffering from incurable cancer who have entered for life. They have been moved into the male surgical ward and into other wards whilst the cancer wing is painted and cleaned, and to attend on these patients a few nurses are of course on duty. But for the others the necessity for a new roof for Middlesex Hospital will mean a splendid rest, and we hope a refreshing holiday.

### RELIEVING OFFICERS AND MIDWIFERY ORDERS.

The President of the Local Government Board was asked by Sir Walter Foster on Monday whether he is aware that in some cases difficulty has arisen from poor women being unable to obtain assistance in their confinement in consequence of unregistered midwives having been warned not to attend such cases, and no others being available. Sir Walter also inquired whether under these circumstances the President would issue an order pointing out to Boards of Guardians the duty of the relieving officer to grant midwifery orders to women destitute of the means of procuring medical attendance. The reply of Mr. Geraid Balfour was that no case of the kind referred to had been brought to his notice, and he promised that if the hon. member would furnish him with particulars of any, he would cause inquiry to be made and would consider what action should be taken. Sir Walter has since sent to Mr. Gerald Balfour the report of a case which came before the Smallburgh Guardians a few days ago. In this instance one of the district medical officers was called to attend a confinement, because, as he said, the women in the district were afraid to go to the assistance of a patient since the Midwives Act came into operation. It will be interesting to see what the President of the Local Government Board has to say in this matter.

### THE TRAINING OF MENTAL NURSES.

NURSES will be interested in reading the account which appears in another page of a significant new departure at Bethlem Royal Hospital. The opening of Barkham House for the reception of paying patients, who are not certified, on terms which are not beyond the means of the well-to-do middle class, is probably the beginning of a great development. It will also be seen that Dr. Hyslop, the medical superintendent of the institution, at whose instance, we believe, the treatment of persons suffering from neurasthenia was decided upon, has visions of a home for the training of nurses. This is a welcome indication that the authorities of one of the greatest and most beneficent of charities are keenly alive to the importance of sparing no efforts to improve the standard of mental nursing.

### EASY CHAIRS FOR NURSES.

THE Huddersfield Guardians discussed at their last meeting the question of expenditure, and the point was raised whether they were justified in buying "beautiful carpets and easy chairs for the nurses." The guardian who brought this subject forward contended that easy chairs are not needed for the nurses to rest themselves in "after a day of doing little or nothing," but he also complained that the medical officer received £20 to give the nurses a few lessons in midwifery, "which had not done them a bit of good." We are not surprised that his views did not obtain much support. We think that it is quite possible to provide nurses with luxuries which the ratepayers cannot be reasonably expected to approve, and we are afraid that in some quarters there has been a tendency in this direction. But easy chairs are, in our opinion, necessary for nurses who work hard all day. The expense, compared with chairs which are not easy, is small, and the advantages are obvious.

### A CHALLENGE TO KENSINGTON.

MISS LOUISA TWINING declares that the amount collected by the Kensington District Nursing Association in subscriptions and donations is not more worthy of the Court suburb than the sum obtained in Paddington for a similar purpose. For such a locality, she urges, £569 is somewhat pitiful, especially as two more nurses are badly needed in North Kensington. Miss Twining makes a couple of practical suggestions—one that prosperous tradesmen should be asked to contribute five shillings a year, and the other that there should be offertories on behalf of the association in every parish in which the association works. Why prosperous tradesmen only? There are plenty of private residents who do not at present help, and who yet could easily afford to give a few shillings a year to such an excellent cause. As to offertories, the more the better, but the clergy have so many claims upon them that they cannot always afford to set apart a collection for the District Nursing Association. Moreover, Kensington is rich enough to find all the money required without appeals being made from the pulpits of the churches.

### CHESTER UNION NURSES.

THE Note in our issue of last week headed "Unfounded Charges against Chester Nurses" should have been applied to another institution in the county where the occurrence reported took place. We very much regret the confusion into which our correspondent fell, and offer our sincere apologies to the staff of the Chester Union Workhouse, and more especially to the master and matron.

### THE BALANCE AT DARLASTON.

IT is the good fortune of the Darlaston District Nursing Institute to continue to possess a very substantial balance of cash in hand. For the year which has just closed the amount of the balance is £460, or £80 more than it was in the previous twelve months. This happy result is largely due to the sum contributed by the workmen in the town. Last year it reached the respectable total of £150. So long as the working men of Darlaston thus rally round the nursing organisation, which is doing much excellent work in the town, there is no danger that it will be crippled by lack of the sinews of war, and we are glad that a proposal which was made at the meeting to alter the rules of the Association, so that the nurses "who have not a great deal to do" might take cases at a fee of 10s., was vetoed. As each of the nurses paid an average of 20 visits a day in June, they have plenty to do, and, apart from this, the innovation suggested is not sound policy. The chairman, in opposing it, remarked that the institution is better off than any other in a town of the same size, and the advice "to leave well alone" was wisely adopted.

### THE "PROPOSED NURSES' EXCHANGE."

AT the last meeting of the Wortley Guardians the question of the proposed exchange of hospital nurses was discussed. The chairman, who alluded to the "Proposed Nurses' Exchange," said that he attended the Wakefield Conference, and as a general scheme he thought that it would work all right. His idea was that a periodical list, containing the names of nurses who could be exchanged, should be sent round to each hospital in the West Riding, that there should be some fixed scale of payment, and that in addition to the salary paid at her own hospital the nurse exchanged should receive a little for the extra trouble. But he was not in favour of the establishment of a central bureau if it meant the payment of any salaries. One of the Guardians, observing that the Wortley Union Hospital has more nurses than patients, maintained that if nurses went away, they would want more money, and he thought that the scheme would therefore involve more expense to the ratepayers. It was decided to discuss the matter further when the committee appointed at the Conference had issued their report.

### UNDUE MODESTY AT COLCHESTER.

It is possible for an institution to be too modest. At the annual meeting of the Colchester Nursing Association the Mayor said that it was so modest that he expected its existence was hardly known to many of the inhabitants. After this statement we are not surprised that, in spite of the fact that it has lived for seven years, it does not receive sufficient financial support. It is the business of the committee of a Nursing Association to take care that its existence is known to everyone in town. If people are not aware that there is such an association, how is it possible for them to subscribe to it? The annual report says that the lady collectors worked hard, but it is clear from results that they do not work hard enough, or that the number of them is insufficient. The hope of purchasing a nurses' home has not yet been realised, and we do not suppose that it will be until a good deal more energy is thrown into the management of the association.

### A DEFICIENCY AT ACCRINGTON.

At the annual meeting of the Accrington District Nursing Association attention was necessarily called to the weak feature of the report. Though the balance in hand at the commencement of last year was £83, there was at the close of the period a balance due to the Treasurer of £6. This is not a position at the end of seven years upon which the town of Accrington can be congratulated. There is room for improvement in another direction. During the 12 months three nurses visited 9,883 cases, or an average of 3,294 each. In other words, 60 visits a week, and the result must be that the nurses are overworked or that their visits are too hurried. Either is bad, and the obvious remedy is the appointment of a fourth nurse. In order to increase the staff, however, the amount received from subscribers should be £300 a year instead of £145.

### FINANCES AT NEWCASTLE.

In moving the adoption of the annual report of the Cathedral Nursing Society of Newcastle-upon-Tyne, the chairman said he thought that it was satisfactory in all respects save revenue. The exception, however, is very important, for the expenditure of the society during the year under review was £1,909 as against receipts £1,512. This is a serious difference, and we are surprised that it was not dwelt upon with more emphasis at the annual meeting. The value of the work of the nurses is undeniable. In the last 12 months they nursed 1,718 cases and paid 17,439 visits. It is also gratifying that the Convalescent Home at Hexham, with a trained nurse in charge, is in full swing, and that the country branches of the organisation are all flourishing. But the Newcastle people ought not to allow the society to be compelled to have a bank overdraft on the revenue account of £238, and an overdraft on the capital account of £332.

### QUEEN ALEXANDRA'S MILITARY NURSING SERVICE.

We are officially informed that Miss M. G. Hill, R.R.C., sister in Queen Alexandra's Imperial Military Nursing Service, has retired on a pension on account of ill health, and that the following have been provisionally appointed staff nurses:—Miss M. Antrobus, Miss E. F. Armstrong, Miss M. Brown, Miss M. M. A. Copinger, Miss M. Davis, Miss H. B. Derby, Miss C. D. E. F. Dunn, Miss E. K. Kaberry, Miss M. L. Kaberry, Miss C. G. Lees, Miss M. L. Macartney, Miss A. C. Mowat, Miss A. A. Steer, Miss A. Ayre, and Miss M. C. E. Newman.

### DEATH OF A SISTER AT SEA.

The death of Miss Annie Fraser, formerly sister at South Devon and East Cornwall Hospital, has occurred under unusual circumstances. Having served in Egypt, she took up a year ago the appointment of sister at the Government Hospital at Accra. Last month she was granted four months' leave of absence and appeared to be in excellent health and spirits when she embarked on the 27th for home on the West African steamship *Zungern.* Three days later she was attacked with remittent malarial fever and though every possible care was given her, she succumbed on the afternoon of July 3rd and was buried at sea on the following day.

### BARRY NURSING INSTITUTE.

For the purpose of welcoming the new lady superintendent of Barry District Nursing Institute an "At Home" was held the other evening, at which a large number of the subscribers were present. An interesting feature of the proceedings was the presentation of several volumes of literature to the late superintendent, Miss Rice, as a token of appreciation and good wishes. Among those who offered her congratulations was the superintendent of the Cardiff Victoria Nurses' Institute.

### NEW HOME AT WEST BROMWICH.

The foundation stone of a new Nurses' Home in connection with the West Bromwich District Hospital, has just been laid with Masonic honours by Lord Dartmouth. The Home is intended to accommodate about 24 nurses, and the total cost is estimated at £3,000. It will be a very commodious and suitable building when it is completed.

### ADELAIDE GENERAL HOSPITAL.

A domestic quarrel at the Adelaide Hospital some time ago resulted in the exclusion of the students from the wards. Harmony having at length been restored, the students have been readmitted, and the nurses, as one of them writes to tell us, have been compelled, to their great disgust, to give up a large number of their most particular dressings to them, besides losing also a number of clinics.

### SHORT ITEMS.

The matron of the Ladbroke Nursing Home, Notting Hill, asks to announce that she has opened a branch at Southend-on-Sea, known as the Ladbroke Nursing Institution and Convalescent Home, 6 Royal Terrace. The house is situated upon the sea-front, at the highest point of the town, close to the pier, and has large secluded gardens in front.

### The Nursing Outlook.

"From magnanimity, all fear above;
From nobler recompense, above applause,
Which owes to man's short outlook all its charm."

### THE NIGHTINGALE SCHOOL.

This School will ever be memorable in the history of nursing from the service rendered by its illustrious founder Florence Nightingale, and the fact that it was the first attempt in the history of the world to train women as nurses for the sick in a systematic, practical way. It is memorable, too, from the circumstance that from first to last the management of this School has been prudent, and cautious, and that those responsible for it have always shown wisdom in the selection and training of probationers, in the management and expenditure of the funds, and in the spirit which they have succeeded in infusing into each nurse who has passed through the school. It is a remarkable fact, too, that those who have most to do with the management of hospitals and kindred institutions, and are in the habit of inspecting them, quickly recognise the Nightingale nurse, when they find her in responsible charge of a modern hospital, from the thorough way in which she grasps the details of each department in her charge, and the uniform efficiency which characterises her control over the whole establishment. No doubt much of the credit for this belongs to the late Mrs. Wardroper and to those who have followed her in the control of the school. Only the other day, when visiting a great hospital, we were struck with the improvements which had been introduced, and were conscious of an active influence for good in each department. A Nightingale nurse had recently been appointed matron of the hospital we refer to with the gratifying results just stated. It is an accepted truth that few things are more difficult in the present day than to find a really able and capable woman, possessed of the necessary qualifications, for appointment to the higher posts in nursing in this country. So long as the present high standard of efficiency is maintained throughout the Nightingale system, this school must continue to occupy the first position in the nursing world.

The report for the year 1904 shows steady progress. Thirty-eight probationer nurses, after completion of one year's training, were placed on the register and admitted to the extra staff of St. Thomas's Hospital in order to complete their training. An important change in the system took place during the past year by the issue of certificates of training in lieu of gratuities to those who had completed their curriculum. Eighteen of these certificates were awarded to nurses admitted after October 1st, 1900, who had satisfactorily completed three years' training and service in the hospital. The wisdom and justice of this new departure cannot be questioned, and it must tend to still further enhance the reputation of the Nightingale school. During the year the important post of lady superintendent of the Hospital for Consumption, Brompton, has been filled by the appointment of Miss A. Lloyd-Still, whose capacity and zeal have already accomplished much. The work which Miss Lloyd-Still has carried out in connection with the opening of the Heatherside Sanatorium for Consumption at Frimley is remarkable. When this is added to the heavy responsibilities devolving upon her at Brompton, Miss Lloyd-Still's great energy and powers of work must be gratefully recognised by everybody. During the year other nurses from the Nightingale School have been selected for appointments to various hospitals and infirmaries in England, Egypt, India, Mauritius, and to Queen Alexandra's Imperial Military Nursing Service.

It is the laudable ambition of many a thoughtful woman who desires to become a nurse to be trained at the Nightingale School. This is not to be wondered at in view of the facts we have just stated. Its popularity in the past has been further due to the circumstance that the managers of the school have always laid themselves out to secure the services of the most capable and experienced matron trained under their own system for the post of superintendent of the training school and matron of St. Thomas's Hospital. This position may be regarded as the blue ribbon of the nursing world. To the poorer probationer there is the added attraction that the minimum salary after the first year of training is £20 per annum. Each nurse has a separate bedroom, and clothing to the value of about £4 per annum. The Nightingale School offers special facilities to gentlewomen, who are admitted to be trained in the practice of hospital nursing with a view to become qualified for superior situations in public hospitals and infirmaries, or in the Army Nursing Service, or for nursing the poor at their own homes under Queen Victoria's Jubilee Institute for Nurses. These gentlewomen must be between 24 and 33 years of age; they receive no salary as a rule, and usually pay a premium. Without payment each candidate has to undergo three years' training; where a payment of £30 is made the obligatory service extends to two years, but this period may be reduced to one year by a payment of £52. We think it well to give these particulars in commending the work of the Nightingale School, owing to the number of inquiries which we receive from time to time.

The managers of the Nightingale Fund show a commendable spirit of fairness to their contemporaries and to all interested in nurses and nursing work. They conclude their report by a statement to the effect that the Royal National Pension Fund for Nurses "affords to nurses a safe means of providing against sickness, accident, or old age."

### Medical Electricity and Light Treatment.

By Kate Neale, Sister-in-Charge of the Actino-Therapeutic Department, Guy's Hospital.

### II.—GALVANISM AND FARADISM.

(*Continued from page* 255.)

### *Electrodes.*

An electrode is the special form of terminal which is attached to the free end of a wire, to be pressed against or passed over the skin of the patient, so conveying the current to him. It is usually made either of metal, sponge, fine wire or prepared carbon, and the shape varies according to the method of use. Fig. 2 A shows the simplest form of Sponge Electrode. It consists of a wooden handle having at one end a screw terminal to which you attach your wire, and at the other a simple arrangement for grasping the sponge, which latter should be about the size of the palm of the hand, of fine texture and free from grit. I should advise you always to use the sponge electrode in preference to any metal variety, unless there be some special reason for doing otherwise; the only precaution you have to take with it is to moisten it well before use.

Fig 2B is a type of electrode used for testing muscles. It is very similar to the one just described, but in place of a sponge there is a piece of prepared

D C B A

Fig. 2.—Electrodes.

carbon covered with chamois leather or lint, which must be moistened before application.

Other forms of electrodes are seen in fig. 2, C and D. Fig. 2 D is known as a Brush Electrode, and does not need moistening. It produces a rather unpleasant and painful feeling, and should be used only on special instruction from the doctor. Sometimes it is desirable to apply an electrode with a much greater surface area than any of the preceding, and then you should use such a one as fig. C. It consists of a sheet of copper with a terminal to receive the wire, and is generally placed in contact with the patient's back. And here note particularly that you must *never apply bare metal to the skin* on any account; if you do, there is a great risk of the current producing a serious burn. Therefore, before using the plate electrode you must wrap it in well-moistened lint, and it is further advisable to shift its position every few minutes.

### *Labile and Stabile Applications.*

When a doctor orders galvanic treatment for a patient, he will always specify some particular way in which he wishes it applied. He may, for instance, want you to fix the electrode to the anode wire and apply that to the diseased part. In other cases he may wish the electrode attached to the kathode wire, and the anode pressed against a healthy part of the body. Or, again, you may be told either to keep the electrode stationary on one area of the skin, or to move it slowly up and down a limb. In expressing his instructions he will make use of certain terms which need explanation.

If he orders *Anodal Treatment* to be used, you will understand that the electrode is to be attached to the anode wire and then applied to the part under treatment, at the same time placing the kathode on some other part of the body such as the nape of the neck. Again, by *kathodal treatment*, he will mean that the kathode wire is to be screwed to the electrode, and this brought in contact with the diseased area, the anode being put at the nape of the neck. Further, if he wishes you to keep the electrode stationary on one area of the skin he will use the term *Stabile Application*, but if you are to move it up and down he will speak of *Labile Application*. You will see that there are therefore four kinds of treatment by galvanism—namely:

1. Anodal Stabile treatment.
2. Anodal Labile treatment.
3. Kathodal Stabile treatment.
4. Kathodal Labile treatment; and you must always be very particular to notice which of these four has been ordered, as each produces a different effect.

### *Methods of Treatment.*

You now have some idea of the appliances used in galvanism, and how to fit them up. The next question is, What are you to do in treating the patient?

A galvanic current is always applied directly to the skin, and never through any garment, so first bare the limb that you are going to treat. Let us suppose you have arranged your instrument for kathodal labile treatment, and the arm is the part you are to operate on. Uncover it so that the sleeve shall be well out of your way. As a rule, it is best to have the whole limb uncovered. Now wrap the anode wire in a sponge that has been well damped in warm water. If you use one that is at all dry the current will experience great difficulty in passing through the skin, because human skin, unmoistened, offers considerable resistance to the flow of electricity, but this defect is in great part done away with by freely damping the sponge. Always remember then to wet your sponge before use, and to remoisten it from time to time, especially if it be in a sponge electrode, which, in labile treatment, soon gets dry. In applications to the head you must as an additional precaution well moisten the hair, to facilitate the flow of current. Wrap the anode wire in a damp sponge, taking great care that the end of the wire does not project, otherwise it will produce a burn where it touches the skin. To avoid all chance of this, use no sponge that has any large holes in it, but select one of fine texture. The wire and sponge constitute what is called an *Indifferent Electrode*, and the next thing is to see that this electrode comes in good contact with the skin. To effect this the patient may grasp it in his hand, if he can be relied upon, but it is often wiser to adopt another method which will be described below. To the kathode wire you have already fixed a suitable electrode, and, holding the handle in your right hand, you press its sponge against the skin of the patient's arm, firmly but gently, and stroke it up and down with the same kind of movement, though not quite as quickly, as if you were painting, taking care the whole time to keep the handle vertical to the surface of the limb.

It is essential to maintain the electrode sufficiently firmly pressed against the skin, otherwise there will not be good contact, and the full effect of the current will be lost. When you have moved as far as necessary in one direction (say from the shoulder down to the elbow) and are about to reverse the movement, take care not to raise the electrode off the skin. If you omit this precaution the patient will receive an unpleasant shock each time the current is broken. Continue this stroking action regularly for as long as the treatment has been ordered—usually from 10 minutes to a quarter of an hour, though the actual time will be specified by the doctor—and then carefully dry the patient's arm.

Should anodal instead of kathodal treatment have been ordered, the procedure differs only in one point, connect the kathode to the sponge the patient holds in his hand and the anode to the electrode. Then apply the electrode (now the anode) exactly as you did the kathode in the previous case.

It is not always necessary to make the patient grasp the indifferent electrode in his hand, and often, especially in the case of a child who would probably loosen his hold long before the treatment was over, it is better to put the sponge, well wrapped round the end of the wire, and covered on the side away from the skin by a piece of gutta-percha tissue to protect the clothes from getting wet, at the back of the neck, and pressed down between the collar or collar-band and the skin. But never, on any account, wrap the sponge up entirely in the tissue because no current will pass through gutta-percha, which is a non-conductor. Another suitable place for the sponge may be found in the bend of the knee, where it can be gripped between the thigh and calf.

When the treatment ordered is "stabile" you connect the patient as before, but the electrode is kept stationary on one area of skin the whole time, or, if the treatment has been ordered to a particular muscle, on different areas of skin over that muscle, for a few minutes at a time. The stroking movement is never used in "stabile" application.

The treatment finished, unscrew the electrode wires and wind each round a pencil, or, better still, a penholder, and then, on withdrawing the holder, the wire will remain in a neat coil ready for future use. By taking this trouble you will probably save yourself the annoyance of finding your apparatus refusing to act because the covering on the wires has cracked and the current leaked. I need hardly say that after using any sponge you must carefully clean it (in weak ammonia or salt and water) before applying it to another patient.

In addition to the above methods galvanism is sometimes administered by placing both electrodes close together on the diseased limb. This is known as the *bipolar method*, as opposed to the unipolar, where the indifferent electrode is on the neck.

In Section VI. you will find an account of yet another modification of galvanic (and faradic) treatment, namely, the electric bath.

Finally, there is one warning I have omitted in the foregoing description, so as to be able to lay greater stress on it here. When your apparatus is fitted up and your patient quite ready for treatment there is a precaution you must always take as a matter of routine, and to which no exception must ever be made. *Before beginning treatment test the current on yourself.* I remember a patient whose treatment the nurse had almost finished casually remarking that he had felt nothing the whole time. The nurse had quite forgotten to switch on the current before she began, and her quarter of an hour's work was wasted. Such a mistake will never occur if you follow out the precaution I have just given, and it may be of service to you in other ways. Sometimes a battery works better than at others, and you may quite unintentionally give a patient a current that is so strong as to be unpleasant, or even painful. This you will always avoid by testing the current on yourself. Again, you will sometimes come across patients—especially children—who complain of the strength of the current in the hope of persuading you to weaken it, but if you are in the habit of trying the current you administer you will soon get to know what is the right strength, and so be able to judge whether the current is really painful or not.

### The Nurses' Clinic.

### THE DISPENSARY. BY A CERTIFICATED DISPENSER.

### MIXTURES.

It would be quite impossible in the limited space of these articles to give the reader all that could be said upon the subject of mixtures, but a few hints may here be given of the general rules and best ways of mixture making. It must first be again and again repeated that however theoretically well-read the student may be, an ounce of practice is equal to pounds of theory. No books that ever were written can give quickness, or neatness and despatch to a beginner—time and practice alone can achieve this. The first thing to know is the order in which substances should be mixed, and in doing this there are many things to be taken into account, namely, chemical reaction or incompatibility colour, and whether a clear or thick mixture will result, also which drugs put together may cause explosions to occur; for instance, turpentine and iodine put together explode, as does creosote with nitrate of silver; in these cases, and in others of a similar character, the disagreeing ingredients must be mixed separately and kept as far apart from each other as

**THE NURSES' CLINIC**—*Continued.*

possible. A gargle frequently ordered is one containing chlorate of potassium and hydrochloric acid, and unless great care is taken an explosion will inevitably result; the pot. chlor. should be put dry into the bottle and the acid added immediately, causing chlorine to be formed, and the bottle should be lightly corked and placed on one side for some time before adding the water, to allow the chlorine to develop. The order in which each particular prescription has been compounded should always be recollected by the dispenser, as in many cases the order in which the ingredients are put together affects the colour of the mixture. A case in point is when quinine sulphate and liq. ferri. perchlor. are ordered together; the quinine must be dissolved in the liquor as it is very insoluble in water, and the way in which this is done makes all the difference. If the quinine is put into the measure glass, and the liquor poured on it, it makes a clear mixture; if, however, the quinine is put into the liquor the mixture becomes thick and muddy, and the patient receiving first one clear, and then a muddy-looking fluid, would complain, very naturally, that they were not the same thing. Another case is where liq. ferri. dialysati is ordered with liq. arsenicalis, each of which should be separately diluted with water, or the mixture will not be clear. Quinine is one of the most troublesome drugs to dispense—it is very insoluble in water, but it is soluble in acids and spirit; if it is ordered without either, a little mucilage may be used to suspend it, rendering it less bitter to the taste. When ordered with bicarbonates, the quinine must be rubbed very fine and the bicarbonate added to it in a state of solution. Quinine sometimes occurs in a prescription with inf. rosæ acid, in which case a copious precipitate of tannate of quinine is deposited, therefore requiring a "shake the bottle" label. In a mixture where the quinine has been dissolved in an acid and tincture of sumbul is also ordered, the latter will cause the quinine to reappear.

Sometimes physicians write their prescription in the order in which they can be compounded, but this cannot be relied upon. It is a good plan to pour the tinctures, etc., as they are measured, straight into the bottle, after first putting in a little of the vehicle, then adding the syrups and essences, and finally filling up the bottle with the water or infusion ordered. One rule is always to put a poison in last, thus minimising the risk of its being put in twice, should the dispenser's attention be unavoidably withdrawn. Ether and any other substance likely to evaporate should also be added at the end.

Nearly all books on dispensing state that distilled water should invariably be used on account of the presence of lime in tap water; this, however, is not at all necessary. In making lead lotion students are always told to use distilled water to make the lotion clear and bright; some doctors on the contrary prefer that tap water should be used for the purpose of making it cloudy, and so not to be mistaken for plain water.

If a salt in excess of solubility be ordered it must be rubbed up as finely as possible in a mortar, adding the water or infusion little by little, thus ensuring that as much as possible be dissolved, the surplus remaining as a fine powder at the bottom of the bottle, and perhaps, on standing, converted into crystals owing to variations of the temperature; a "shake the bottle" label must always be affixed in a case of this kind. The solubility of salts is increased or diminished by the presence and contact of others. Hence all salts ordered in one mixture should always be rubbed up together to increase solubility. Iodide of potash increases the solubility of all mercurial preparations and of iodine. Citrate of potash helps salicylic acid to dissolve; the latter also dissolves very readily in a solution of acetate of ammonia forming salicylate of ammonia and setting free acetic acid. Salicylic acid is almost insoluble in water, about 1 in 550. Salicylate of soda (sol uble one in one) is frequently used instead. Spirit of nitrous ether is incompatible with all salicylates.

Alkaloids are not soluble in water, so an acid must be added to form a salt which will then be soluble about 1 in 20. That alkalies precipitate alkaloids is a rule always to be remembered, therefore they must be kept apart as far as possible. To distinguish between an alkali and an alkaloid is sometimes of some difficulty to a beginner; an alkaloid is always an organic substance; it turns red litmus blue, and combines with an acid to form a salt which is generally easily soluble in water. The English names of alkaloids always end in "ine," as atropine, caffeine, strychnine, etc. If a substance be tested to see if it is an alkaloid, but does it not form a salt, it may be a glucoside. Glucosides undergo hydrolysis (or decomposition) on contact with free acids, and form a muddy precipitate.

When vegetable substances, wholly or partly soluble in water, especially such as contain tannin, are ordered to be mixed with metallic or earthy salts, they must each be separately dissolved in a large portion of water.

The scale preparations (Ferri. et ammonii citras, ferri. et quinine citras and ferrum tartaratum), so-called on account of their physical characteristics, must either be dissolved in a mortar with warm water, or put into a bottle half full of water (or other vehicle) and well shaken. If put into a dry bottle they stick to the sides, and when the water is put in they cake at the bottom, and are extremely difficult to dissolve. They must always be put into a mixture before anything else. When ordered in an effervescing mixture a scale preparation should always be put into the acid solution. Ferri. et Ammon. Cit. with a bicarbonate causes effervescence, and the mixture should stand uncorked for a short time to allow the escape of the $CO_2$. Ferri. cit. et strychninæ is also a scale preparation, but seldom used on account of the strychnine separating out.

Another substance which is sometimes a source of difficulty to an inexperienced dispenser is bismuth. The carbonate of bismuth is best suspended by about half its own weight of pulv. trag. co., or more may be used, but there will always be a heavy precipitate. If mucilage of acacia or tragacanth be used it is likely to form an indiffusible mass. When bismuth subnitrate is ordered with bicarbonate of soda or potash, effervescence occurs and $CO_2$ is given off. This is another case where the bottle may be burst by the mixture exploding if corked up before the chemical action is complete. The reaction may be hastened by trituration in a mortar with hot water.

A good deal of trouble is often caused by the froth upon mixtures after a bottle has been shaken, thereby preventing the whole of the vehicle being added, this is obviated by the addition of a few drops of tincture which may be reserved for the purpose, or a little spirit if allowed. Vegetable solutions are very apt to cause froth when shaken.

When a mixture is ordered containing carbonate of magnesia some water should be put into the measure glass and the magnesia added; if water is put on to the magnesia, the latter rises to the top and is very difficult to disperse. This also applies to salicylate of soda which is likewise very light. Preparations containing resins with little other dissolved material turn milky on the addition of water, and the active substance is precipitated, as tincture of cannabis indica—they should therefore be suspended with a little mucilage. If the active constituents are soluble in water, they form a clear mixture and do not require a mucilage, as for example, tr. opii.

Fluid extracts containing resinous bodies, tinctures of gum resins, and aromatic or resin—containing drugs which are made with the weaker alcohols are not supposed to require a mucilage, especially as they are generally prescribed with other ingredients containing extractive or saccharine matter, and if an alkali be present it helps to emulsify, but if it be found that a better mixture would be formed by the addition of a little mucilage it is better to use some. Tincture of myrrh does not require it. In dispensing resinous fluids in aqueous mixtures mix everything with the water except the resinous fluid, which add last and gently shake. Some extracts form a good substitute for mucilage.

### The New Departure at Bethlem Royal Hospital.

### THE NURSING OF NEURASTHENIA.

A VERY important new departure and one that may have far-reaching consequences, has been made under the auspices of the Governors of Bethlem Royal Hospital. They have converted Barkham House 60 and 62 Lambeth Road, into a residence for paying patients who are not certified, but who are suffering from neurasthenia and other mental and bodily ailments, which, in default of careful nursing and medical treatment, may develop into insanity. By the courtesy of Dr. Hyslop, the medical superintendent of Bethlem, I had an opportunity the other day of visiting Barkham House and of obtaining some interesting information respecting the undertaking.

As Dr. Hyslop explained, it has not been hastily launched. So far back as October 1902, the idea first occurred to him that it might be a boon to a considerable section of the community to provide homes for patients suffering from neurasthenia, nervous debility, and other mental ailments—persons, in fact, on the borderland. The question was brought before a special Court of Governors in the same month, and it was finally decided to make a start. Before opening, however, a great deal had to be done, many formalities to be observed, and the houses to be used for the experiment to be not only equipped, but practically reconstructed and almost rebuilt.

"The Governors of Bethlem," said Dr. Hyslop, in course of conversation, "in discussing the question were mainly influenced by the knowledge that all the Homes in London, especially those at the West End, are beyond the resources of most people, and they came to the conclusion that by adapting some of the houses on their property immediately facing the Hospital, they could receive such patients at the limited payment of four guineas a week at the outside."

"Only ladies, I think?"

"Yes, only ladies for the present, at any rate. We were not able to get the two houses in Barkham Terrace ready until the early part of the present year, and since then we have had several applications from persons who are not certifiable. It is not the object of the Governors to make any kind of profit, and the inclusive terms I have mentioned were really fixed by the Charity Commissioners."

"And the administration?"

"It is carried on by the physicians of Bethlem Hospital, the steward, a specially-appointed matron, and by fully-qualified nurses trained at the hospital."

"In other words, the patients will have the opportunity of obtaining the benefit of the experience gained by those in authority at Bethlem?"

"That is how the Governors regard it, and they believe hat the new institution will supply a very distinct want. We think that it ought to be utilised by physicians who feel the responsibility of keeping borderland cases in private homes, for under the regulations drawn up by the Governors of Bethlem the various medical men who send patients to Barkham House can still keep in touch with them, while the responsibility for their welfare is placed upon the physicians at Bethlem."

"I should like to know about the nursing?"

"The nurses have been specially trained for the treatment of these cases, and hold certificates of efficiency from the Medico-Psychological Association. They thoroughly understand the various forms of electrical treatment, massage, etc. It is, in fact, one of the great advantages offered to persons who are nervously and slightly mentally affected that they have the care of those who are entirely conversant with all forms of mental and nervous ailments. Bethlem, it must be remembered, is a charity managed by a large and very influential body of governors. The new venture is well within their sphere of doing good, and in this case it is intended to benefit persons of moderate means. If it is appreciated and is used by the public, other houses in the terrace will be employed for the same purpose as they become vacant."

"There could not be a more pleasant outlook," I observed as we walked past the lawns at Bethlem and proceeded across the road to Barkham House.

"No; and, so far, the tennis and croquet-lawns of Bethlem have been available to those ladies at Barkham House who have wished to use them. This is a privilege scarcely possible in any other home of the same description in any other part of London."

The matron then conducted us over the house, which has been furnished and fitted up in the most comfortable, tasteful, and complete manner at very considerable expense. There will be accommodation for 20 patients, but the possibilities of the future may be judged by a remark of Dr. Hyslop, who, indicating the ground at the back of the terrace, said that eventually he hoped that the site would be occupied by a home for the training of nurses.

"What would be the maximum number of nurses required?" I asked.

"Eight or ten, according to the needs of the patients. As you see, there is a nurse's bedroom adjoining the bedroom of each patient, and a dining-room for the exclusive use of the nurses is provided. The duties of the nurses are not heavy, but it is essential that they should be bright, intelligent, tactful, and well educated."

"And the rules for patients?"

"They are required to conform to the wishes of the resident physician with regard to visits to and by friends, the taking of exercise, attendance at places of worship and amusement, and generally in all things pertaining to their life in the House. Of course, they are not allowed to bring in alcohol or drugs, and the resident physician determines all questions respecting diet and the use of alcohol. I need scarcely add that a patient is free to leave when she chooses, but the recommendation is to remain as long as it is considered necessary."

The equipment of the House has been most carefully thought out from the block floor in the light and airy basements to fire-escape at the top, which in case of fire insures egress along the roofs of the terrace.

### To Nurses.

**WE invite contributions from any of our readers, and shall be glad to pay for "Notes on News from the Nursing World," or for articles describing nursing experiences at home or abroad dealing with any nursing question from an original point of view, according to length. The minimum payment is 5s. Contributions on topical subjects are specially welcome. Notices of appointments, letters, entertainments, presentations, and deaths are not paid for, but we are always glad to receive them. All rejected manuscripts are returned in due course, and all payments for manuscripts used are made as early as possible after the beginning of each quarter.**

### A Book and its Story.

### RUSTIC ROMANCE.*

"A Village Chronicle," is the name given to a collection of short stories by Mrs. Macquoid, in which a series of incidents, more or less connected, although utilised separately, are woven into tales of a village presided over by a benevolent rector and his wife, who is the guardian angel of all who are in distress. She acts as interpreter of the rustic dramas which opens with her own love story.

"I was an orphan without brother or sister; I belonged to nobody except my great-aunt, old Mrs. Lambert. I lived with her in the west of Ireland in an old manor house called Kinrara. This sounds dull, but I was always as gay as a lark, and usually had my own way with everyone. . . . At Kinrara were dear beautiful woods; above all there was the sparkling river that parts the Kinrara property from Wide-Water, which belongs to Lord Ulster." The day arives when Nora has to confront the unpleasing prospect of being sent away to school. Before going away Mrs. Lambert sends for her, and in a final interview held in the small library at Kinrara she tells her of the plans for her future. "The small library led out of the big library, and was my great-aunt's especial den; no one might go there unless sent for. I have learned since that she was eighty on the day she sent for me. The colour of her hair always puzzled me: it was a dull red, all ends, as it stuck out beyond the edge of her white lace cap. She was tall and very thin. I sometimes thought she did not wear any petticoats under her straight-falling dove-coloured gown; round her throat was a lace ruffle in place of a collar; the ruff was so full, and she held her head so stiffly, the starched points of lace must have pricked her. Though she never praised me, she did not scold me; I thought she was a very nice old woman. To-day her dark eyes looked kindly at me." The object of Mrs. Lambert's summons to her reat-niece was to tell her the conditions upon which she intended to ma Norah her heiress. he desired to unite the Kinrara est the adjoining property of Wide-Water. To effect this Norah must marry Lord Ulster's nephew and heir, Bryan Curragh. The idea was one that met Lord Ulster's wishes also. "Do you understand, child?" she continued. "It is proposed when you are both old enough that you and Bryan shall marry. You see, it is quite simple, Norah." To Norah the project, naturally, seemed the reverse of simple—in fact ridiculous—and had she not promised both of the boys to keep single for each of their sakes? Before leaving Kinrara for school Bryan Curragh appears on the scene. He meets with scant courtesy from the wild Irish girl, whose beauty was well known to many youthful admirers in the vicinity of Kinrara, and who resented the intrusion of a stranger into the little circle over which she reigned supreme. "I did not see Bryan Curragh again. I told my aunt I hated him, and she scolded me; a few days after I was sent to school in Jersey. I stayed there five years."

So ends chapter one of Norah Lambert's romance. Her school life in Jersey was a pleasant contrast in the matter of companionship of her own sex to that of Kinrara. Mrs. Lambert died soon after her great-niece left her, and in the happy days in Jersey the time passed rapidly until her nineteenth birthday came, and with it a summons to go to Paris from a married sister of her father's.

"They had been living for some years in Vienna, as Monsieur de Wazincourt was attached to the Embassy; I had never seen him or my aunt either. I knew she was the Vicomtesse de Wazincourt, and I felt nervous at the thought of living with such grand people. . . . When I reached Paris an old servant met me at the station and took me to my aunt's flat, not very far from the Tuilleries. I found that she and her husband were just going off to their country house about twenty miles off, and they took me with them." My aunt knew a few old ladies staying in our quiet Norman bathing-place; Monsieur de Wazincourt, our only gentleman, soon went off to Brittany to shoot. . . . Before their return to Paris life began anew for Norah.

"The Viscountess's rooms in Paris were in the best quarters. They were fashionably decorated: tapestry of great value, rugs and curtains, exquisite drawings and figurines, embroideries, art work in ivory, metal, china, and curios filled me with delight and wonder. Madame de Wazincourt was very kind and affectionate, and always courteous and gentle to everyone; her charming air of distinction gave her much prestige." Norah found two disturbing elements to her complete happiness, in the absence of fresh air indoors and the presence of a certain Mademoiselle de Croy whose sour pinched face and fawning manners were a constant source of irritation to her. Then her charming bedroom had the drawback of communicating, as is so often the case in Continental apartments, with adjoining rooms. Norah's bedroom led out of that of Madame de Wazincourt's, and consequently Norah had little privacy. She was often, too, an unwilling auditor to conversations between Mademoiselle de Croy and Madame de Wazincourt. One day she overhears a discussion between her aunt and Mademoiselle relative to her marriage. Madame de Wazincourt declares her intention of looking out for a suitable husband or her *protégée*. In reply to some acrid remarks from Mademoiselle de Croy upon Norah's chances, her aunt replies, "I intend to marry Laura as soon as possible . . . but Norah is not a French girl, so I intend that she shall choose for herself. I chose my husband, and he made me happy. Why should I not give Norah the same freedom?" Murmurs of dissent were heard and the discussion ended.

Before introducing Norah to Parisian society much kindly thought was exercised by Madame de Wazincourt on the toilettes in which she should appear to the best advantage. When these necessary preparations were completed and her *protégée* was anticipating the delight of her first ball, Madame approached the subject which she had at heart—the marriage of Norah. Norah then learns that her great-aunt had left directions that Madame de Wazincourt was to have charge of her on the condition that she managed a marriage satisfactorily. Norah had beauty and charm, and, in addition, she was an heiress. As is usual in France, negotiations were opened by her guardian with eligible *partis* who were contemplating marriage. At once three desirable proposals were made, and not until then did Madame divulge Mrs. Lambert's intentions in giving her into her charge. Laura is sent for, and her aunt tells her.

"The business I have to discuss with you, Norah," she said, looking keenly at me, "is the choice of your husband I have already received three proposals for you." So she is taken into the swing of the season in Paris, is introduced to the three suitors, none of whom please her in the least, for she has been attracted by some one outside her circle of partners one evening at a ball. Later she sees him talking to Madame de Wazincourt, and afterwards he comes forward to make himself known to her as her youthful admirer, Bryan Curragh. Lord Ulster, his uncle, having married again and having an heir, the arrangement to unite Mrs. Lambert's property by his marriage to Norah is cancelled, and Norah and he were free to choose as they pleased. How they acted, now they were free agents, will be seen in the story, which is one of the best in a collection which is, on the whole, excellent. "A Village Chronicle", is just the wholesome sort of book for girls of sixteen and others who like to renew the past in records of events which come into the lives of most of us.

* A Village Chronicle. By Katherine Macquoid. (Digby, Long and Co.) 6s.

### The London Hospital.

### OPENING OF THE NEW MATERNITY WARDS.

A further extension of the London Hospital was inaugurated on Monday afternoon by the opening of the new maternity wards. The Countess of Pembroke and Montgomery was to have declared the wards open, but unfortunately she was prevented from doing so by an attack of influenza. Her place was kindly taken by Katharine, Duchess of Westminster.

### Mr. Holland's Speech.

The Honourable Sydney Holland, Chairman of the Hospital Committee, gave a brief sketch of the inception of this new branch of work. He referred to the Midwives Act and to the shortage of qualified women, which would be felt in the future if steps were not taken to increase that supply. The establishment of these wards, therefore, would not only enable them to be of service to lying-in women, but it would also, they calculated, put them in a position to send out some 40 to 50 qualified midwives every year. This might not seem to some a large number, but the organisation required to give them the needed training was very considerable. The London Hospital was obliged to do its duty, and it was a remarkable fact that when they set their hands to a piece of work, the money was always forthcoming. They required £20,000 to establish these wards, and they appealed to the women of England. Fifty thousand personal letters were sent to women, and five out of every 100 recipients answered. The sum of £14,000 was received. They had since obtained the remaining sum needed, with the exception of £500, which he trusted might be forthcoming that day. But the man who had really called the wards into being was Mr. James Hora, who had given £10,000 towards their endowment, in memory of his wife, Marie Celeste, after whom the wards are named. Mr. Holland read passages from a letter from Mr. Hora, expressing regret at his inability to attend the opening, and forwarding another £1,000 towards the endowment, The yearly income required Mr. Holland continued, would be, £1,000, so that Mr. Hora's gifts were, indeed, welcome; and this particular day, he pointed out, did not only mark the opening of the new wards, but was also the 25th anniversary of Miss Lückes' coming to the hospital as matron. It was impossible to speak too highly of her great work, and, as a small appreciation, the Committee had decided to call the new Nurses' Home, shortly to be opened, after her.

### The Bishop's Tribute to Miss Lückes.

The Bishop of Stepney said he felt that he might thank the London Hospital for starting this work in the name of vast multitudes in East London. He welcomed it, not only as affording such immense physical relief to poor women in the greatest crises of their life, but also as being the probable means of leading them to better ideals, since the time of motherhood was perhaps the most impressionable period in a woman's existence. He realised how needful it was that midwives should be thoroughly trained, and the immense advantage of their being connected with a large hospital. In referring to Miss Lückes' splendid work, he said it was always a pleasure to him, as he passed the gates of the hospital, to think that her great personality was spreading its influence over everybody and everything within.

Katharine, Duchess of Westminster, expressed the pleasure which she felt in being of any service to the hospital in coming to open the wards that day. She realised fully, from her knowledge of better conditions prevailing in village places, the enormous need for helping the women of London. She then declared the wards open, and after prayers had been offered by the Bishop of Stepney, asking for God's blessing on the new work, the company adjourned to inspect the wards.

### The New Wards.

They consist of three separate wards, about 20 feet square, for three beds each, with a cubic capacity of about 1,650 feet per bed. At the foot of each bed is slung horizontally a smal cradle for the baby. There is one smaller ward for a separation case. The wards open out on to a pleasant balcony overlooking the hospital garden, so that they are extremely quiet. There is a labour-room, 20 feet square, with a large bath-room adjoining. A scullery, sink-rooms, testing lobby, linen-room, and a sitting-room for the sister-in-charge complete the suite. The whole department is lined with opalite of a delicate green colour, the gift of Mr. Hora, and the labour-room, bath-room, and sanitary annexe have mosaic floors. The doors are of "hospital" type, in teak, being plain slabs without panels. The labour-room has a large bay-window facing north and is specially ventilated, the air being drawn into the room by means of a fan through a gauze screen, and warmed by passing through a copper swing steam coil. The vitiated air is expelled by means of a fan on the opposite side of the room. The whole of the fittings to the sinks, etc., in this room are nickel-plated, to save the labour of cleaning.

The nurses' rooms are on the floor immediately above the wards. A large part of the maternity work will be done in the women's own homes, under the superintendence of two sisters. Provision is made for the training of 12 midwives. The midwives will spend one month in the wards and two in the district. All the worst cases will be sent to the wards. A special feature is that only nurses who have been fully trained for four years at the London Hospital are allowed to qualify here as midwives.

### Infirmary Nurses' Rations.

That endlessly-vexed question, the food of the nurses, has come up again, this time in connection with the Poor-law Hospital at Halifax. A new scale of diet was brought forward and adopted, though not without some discussion. In this the allowance of meat for all officers is reduced: that for the medical officer and matron from 7 lb. to 6 lb. a week, and for nurses from 5½ lb. to 5 lb., while, when poultry is served, a chicken is to suffice for four people instead of three—a change which will at least render the carver's task easier The number of eggs is reduced from seven to five per head a week. These economies can be effected without diminishing in the least the comfort of the officers, and will lessen waste and diminish opportunities for theft. The allowance of tea remains at ½ lb. per head a week, a quantity which, if indeed the officers receive it, seems excessive. One cannot but wonder if the Local Government Board had the benefit of the advice of a practical housekeeper when they sanctioned some of the dietary tables for different unions. In at least one metropolitan union the medical officer and matron of the infirmary are allowed 14 oz. of tea each, a weekly amount which, if they consume it, would be rather injurious to them. It seems strange also to assume that tea-drinking capacity increases in direct proportion to a rise in rank, for in this institution the nurses receive, as at Halifax, 8 oz. a week. As a matter of fact, an allowance of 6 oz. per head a week for all ranks would be an ample supply, erring, if anything, on the side of lavishness. In one respect, however, the economy of the Halifax guardians is to be regretted. Hitherto fruit has been served three times a week; now the rule is to be that fruit to the value of 6d. a week is to be allowed for matron and medical officer, and 3d. for nurses. The principle of fixing an entire dietary scale by cost instead of by exact measurement is a sound one, but not that of settling one article in this fashion. Nurses, coming from their work in the wards, often lacking in appetite from sheer weariness, would find fruit more appetising than many other articles of diet, and for indoor workers it is as wholesome as it is refreshing. Doubtless guardians, as large customers, can get better value for their money than retail purchasers, but allowing for that, the allowance of something under ½d. a day will not give much to each. Even those who are far from being vegetarians are more inclined than they were in former days to put a high value on fruit as an article of diet, and in this matter the Halifax guardians might show a little more generosity to their nurses.

### Everybody's Opinion.

[Correspondence on all subjects is invited, but we cannot in any way be responsible for the opinions expressed by our correspondents. No communication can be entertained if the name and address of the correspondent are not given as a guarantee of good faith, but not necessarily for publication. All correspondents should write on one side of the paper only.]

### A WARNING TO NURSES.

"A Leyton Nurse" writes: I wish to warn nurses against lending money. A short time ago a woman, dressed like a lady, called upon me and stated that she required a nurse-attendant. I lent her some money, and then found that the address she gave me was false. Of course I have never heard any more of her.

### NIGHT NURSES AND LITTLE COMFORTS.

"M. F. M." writes: I wonder if other nurses agree with me that when a nurse is on night duty from 8 P.M. until 8 A.M., and is in the wards the whole of the time, she needs a little comfort; as, for instance, a cloth on the table on which she has her meals, and to make the kitchen look more cosy. There are cloths for the ward tables, but in this particular ward they are locked up at night by the sister-in-charge. I am a certificated nurse, and would like to have other nurses' opinions.

### LEICESTER INFIRMARY.

Miss Rogers, matron of Leicester Infirmary, writes: I shall be much obliged if you will correct an announcement which appeared in The Hospital of July 8th, to the effect that Miss Clarke had been appointed matron of the Infirmary, Leicester. Miss Clarke has been appointed matron of the Poor Law Infirmary. The Leicester Infirmary is the County Hospital, of which I am still matron.

[The appointment of Miss Clarke as matron of Leicester Workhouse Infirmary was announced in our issue of July 1st. In our issue of July 8th a letter headed "Leicester Workhouse Infirmary" appeared referring to this appointment. There is therefore nothing to correct.—Ed. The Hospital.]

### DISTRICT MIDWIFERY AND THE MIDWIVES ACT.

"Futurist" writes: I venture to send you a few words in connection with district midwifery and the Midwives Act. It is generally acknowledged that the Midwives Act has opened out a new sphere of labour for our educated and younger women, especially trained nurses. At the present time the demand exceeds the supply, although there are many qualified and clever midwives who would be glad to hear of a genuine opening for work In many of our small towns and rural districts the untrained but registered woman is willing to take a course of training if an outsider would step in and bear the expense. There is, in these days, a great tendency to pauperise the poorer classes by giving them everything for nothing. Experience has taught me that the lower classes of the community consider it lawful and right to ask for food, clothing, nursing, attendance, etc., and if refused they consider themselves badly used. With this tendency to pauperism, shall we not ask ourselves the question, "Is it wise to provide midwives for the poor at a very nominal cost, or perhaps no fee at all?" I am referring to charitable institutions and nurses supported by public contributions. The public, we all know, count upon these charitable organisations to relieve the needy poor in the hour of sickness. This sickness is unforeseen; a midwifery case is not. Although aware that a nominal fee is charged, there can be no doubt that the charity will be greatly imposed upon, both by people who can afford to pay, and by those who never intend paying. Thus an honest midwife working on her own account is deprived of a living. I am not referring to our large cities of over 500,000 inhabitants, where pupil midwives are trained in connection with lying-in institutions, but to the supply of our rural districts and country towns. Why cannot suitable women be trained for midwifery to work on their own account, and so make the public feel that they must bear their own individual burdens, instead of depending upon a charitable public to supply a need they have hitherto supplied themselves?

### NURSING THE SCHOOLBOY.

"J. C." writes: I have been much interested in reading the articles on Nursing the Schoolboy, and I think the Denstone College Sanatorium an ideal home for sick boys: I hope that there are many more like it. I think the boys in this school, where I first began nursing them, are much the same as the Denstone boys, but the arrangement for their comfort when sick is very different. By way of contrast I would like to describe them. When I arrived I was shown the day and night sick-rooms. The day room was next my bedroom, and I think originally it had all been one, as the window was partly in my room and partly in the sick-room. It was a narrow room with door and fireplace at one end and the window at the other; the floors were bare, the walls white-washed, with some wretched-looking pictures, the glass over them being for the most part broken. A bedstead in the corner near the window, six Windsor chairs arranged along one side of the room, an armchair to match, a bed-chair, a table with a washed-out cover, a small table that reminded me of a litany desk, and a small square of bright patterned carpet near the fire-place, summed up the rest of the fittings. The night sick-room was even more bare, only having two chairs, a bed, wash-stand, etc., and was shut off by a door on the landing. I never could arrive at any conclusion why the rooms had been so arranged, as it was impossible for the nurse to hear a boy so far away. Whenever I could I always put the boys to sleep in the day room. On one occasion I was especially thankful that I had done so for the boy walked in his sleep. The worst feature of the rooms was that they faced the north, so they never had a gleam of sunshine, and they looked on to a back yard where there was nothing to see, though being near the kitchen, unfortunately a good deal to hear. Then in the grounds there was a house known as the "san," where boys who were likely to be ill more than a day or two were sent. Here, though, they had the advantage of the sun. The rooms were the barest and the most uncomfortable possible; any workhouse I have ever visited had a more cheerful look than our "san." I think that, as a rule, the boys were glad to return to school as soon as possible, so perhaps it was an advantage not to make them too comfortable. I hold that all rooms, especially those used for the sick, ought to have the sun some part of the day with plenty of fresh flowers, and I find boys are quite as fond of pretty surroundings as their sisters are, and often more easy to please.

### Presentations.

Mount Vernon Consumption Hospital, Hampstead, N.W.—On retiring from the matronship of Mount Vernon Hospital Miss Longstaff was presented with a handsome travelling clock by the Committee of Management. The nursing staff presented her with a selection of Mr. Rudyard Kipling's works, and the servants gave her a gold brooch. Miss Stevens, who relinquished the post of home sister at the same time, received from the nursing staff a case of silver toilet articles, and from the servants a silver-mounted miniature timepiece.

### Appointments.

No charge is made for announcements under this head, and we are always glad to receive and publish appointments. The information, to insure accuracy, should be sent from the nurses themselves, and we cannot undertake to correct official announcements which may happen to be inaccurate. It is essential that in all cases the school of training should be given.]

Aylsham Workhouse, Norfolk.—Miss Gertrude Hall has been appointed superintendent nurse. She was trained at Chorlton Union Hospital, West Didsbury, Manchester, and has since been head nurse at Thetford Union Infirmary.

Canterbury Workhouse Infirmary.—Miss Kate Mawson has been appointed superintendent nurse. She was trained at Leeds Union Infirmary, where she has since been sister.

Friedenheim Hospital, Swiss Cottage, N.W.—Miss B. J. Bourne has been appointed charge nurse. She was trained at the Royal Albert Hospital, Devonport, where she has since done private nursing.

Lewisham Infirmary.—Miss Hannah M. O'Connor has been appointed sister. She was trained at the Royal Portsmouth Hospital, and has since been sister in the Army Nursing Service and at the Essex and Colchester Hospital.

Montgomeryshire Infirmary.—Miss Annie Easton has been appointed matron, and she will also have charge of the dispensing for the in- and out-patients. She was trained at the Manchester Royal Infirmary and has since been sister at the Greenwich Infirmary, night superintendent at Shoreditch Infirmary, and superintendent nurse at Walsall Infirmary. Miss Easton holds the Apothecaries' Hall certificate.

Paignton Cottage Hospital.—Miss L. E. Penwell has been appointed staff nurse. She was trained at Tiverton Infirmary, where she has since been staff nurse.

Stroud General Hospital.—Miss Ruth Jones has been appointed charge-nurse. She was trained at the General Hospital, Birmingham, and has since been staff nurse in the same institution and night sister at the Royal Alexandra Hospital, Brighton.

### Novelties for Nurses.

(By Our Shopping Correspondent.)

### FOR USE ON A HOLIDAY.

Messrs. Anderson and Anderson, of 37 Queen Victoria Street, and 58 Charing Cross, are issuing a summer holiday circular enumerating many of their useful india-rubber commodities of interest to the holiday-maker. A waterproof ground-sheet strikes one as calling for attention from nurses about to accompany patients to the seaside or to a country resort. The ground-sheet is made of a loose check material in various colours, with a waterproof lining. It can be used on the journey as a hold-all or rug, and at all times it has a variety of uses. The best quality only costs 6s. 9d., and the cheapest sheet is 4s. 9d. There are many other attractive items for seaside use in the catalogue, such as paddlers for small children, bathing caps, and swimming collars. For the cyclist and pedestrian, yachtsman and sportsman, all manner of suitable rainproof clothing is provided. I have not space to enumerate one-half of the useful items of the list, and therefore I recommend my readers to write to Messrs. Anderson for a catalogue.

### CHOCOLATE AND COCOA FOR THE TRAVELLER.

The wise traveller is he or she who provides against those unforeseen events which have a way of upsetting the most carefully thought out plans. The pedestrian and cyclist is especially prone to become a victim to the vagaries of fortune. A late arrival at some resting-place may be a small misfortune, but when the pangs of hunger are added to disappointment the matter is more serious. Therefore, those who are wise provide against such a mishap, and never venture afield without provisions. Messrs. Cadbury have reminded us that their excellent chocolate and cocoa specialities meet the requirements of every holiday-maker. Their milk chocolate is a form of concentrated food of the highest nutritive value, and excellent to allay hunger. It can be carried without adding a perceptible burden to the travelling kit. A tin of concentrated cocoa, or of the still more nourishing malted cocoa, renders the traveller independent of the unskilled and uncleanly cook. All that is needed to complete a repast is some bread and a little milk. These are nearly always obtainable when the larder is otherwise bare. Neither chocolate nor cocoa are spoilt by keeping, and no harm is done if the need for them does not arise on the journey. They can be stored for use on another occasion.

### NURSES' OBSTETRIC BAG.

A very successful maternity bag has just been constructed by Messrs. Arnold and Sons to comply in every respect with the conditions enforced under the regulations of the Central Midwives Board. The bag itself is made of solid calf, and appears most strong and durable both in workmanship and design. By no means one of the least of its innovations is its ingenious lock-joint, which is so constructed that by the mere touch of a finger it holds the bag firmly and widely open while the nurse removes her appliances in comfort unhampered. A removable lining is another advantage. Amongst the bag's actual contents is the syphon vaginal douche, in connection with which I have to mention four commendable specialities. The tubing bears a small nickel-plated fixture, by the pressure of which the flow of water can be stopped and regulated at will. Close to the end to be placed in the basin is a vulcanite appliance—a little semi-circle—which, placed over the rim of the vessel, is most useful in keeping the tubing from slipping. The third improvement consists in the sinker being made of a metal which under any circumstances is guaranteed not to corrode; and, lastly, there is a neat waterproof bag which provides a safe and effective covering for the douche.

The catheter, enema, thermometers (clinical and bath), and other ordinary adjuncts of a midwife's bag leave nothing to be desired in respect to general excellence. With regard to the binder it seems very good and durable. Pins, safety pins, and thread have been provided. The little metal boxes for holding cyanide gauze, lard, powder puff, gauze for plugging, and one with two compartments containing a nail-brush and small square of yellow soap, are all very satisfactory, and, besides their utility, give a neat appearance to the bag. The same can be said of the four bottles, in metal cases with spring tops, which have the names of their contents—ergot, castor and olive oils, and sal volatile—plainly engraved on them. Two bottles of, respectively, biniodide of mercury soloids and boric acid soloids, have also been provided; but with regard to wool, although a neat waterproof bag is enclosed to hold it, the nurse must personally purchase what she requires. It is thoughtful of the manufacturers to include one empty bag in case the nurse should require it for any emergency. The new battiste mackintosh, in spite of its apparent fragility, will not only stand all ordinary disinfectants, but will even bear the test of boiling. The price of the bag is £3 12s. 6d. Nurses' initials will be stamped on free of any extra charge.

### Notes and Queries.

### REGULATIONS.

**The Editor is always willing to answer in this column, without any fee, all reasonable questions, as soon as possible.**

**But the following rules must be carefully observed.**

1. **Every communication must be accompanied by the name and address of the writer.**
2. **The question must always bear upon nursing, directly or indirectly.**

**If an answer is required by letter a fee of half-a-crown must be enclosed with the note containing the inquiry.**

*Lost Property.*

(129) Will you please let me know what steps I can take to recover a bag of laundry left at the nursing institution where I was staying. It was placed where we were told to leave soiled linen, but the laundry where I was told it had been sent deny all knowledge of having received it. Can I legallyclaim damages? It was worth at least 30s. ?—*Sister Grace.*

We never advise nurses to go to law if they can possibly avoid doing so. But if after an appeal to the matron you still wish to do so consult a dependable solicitor.

*Convalescent Homes.*

(130) Will you kindly give me any addresses you may know of convalescent homes where nurses might stay who are requiring rest and change of air, preferably at the seaside and on the South Coast?—*A Tired Nurse.*

House of Rest, Burlington Place, Eastbourne; apply Miss Mason, 10 Finchley Road, London, N.W. G.F.S. Home of Rest, 15 St. George's Terrace, Brighton. St. Mary's Convalescent Home, Eastbrooke Place, Dover. Mildmay House, Y.W.C.A. Folkestone. Meyrick House, Littlehampton; apply Sister Superior, 21 Drayton Gardens, London. S.W. Y.W.C.A. Home of Rest, 2 Liverpool Terrace, Worthing.

*District Nurses.*

(131) Can you tell me where I can obtain particulars of nurses trained in cottage hospitals for district nurses' work? Also where I can obtain information respecting Queen's Jubilee nurses?—*M. L. L.*

Write to the Holt-Ockley Nursing Association, 12 Buckingham Palace Road, S.W. For particulars of Queen Victoria's Jubilee nurses write to the General Superintendent, 120 Victoria Street, London, S.W.

*Nursing Home.*

(132) I am desirous of establishing a nursing home or home for invalids. Could you tell me what neighbourhood about London would be the most suitable for this purpose, or would such a home be better in a seaside place? I am not a trained nurse, but both myself and my sister are good nurses, and my husband for many years has been practising in the medical profession, but had to give up last year owing to ill-health.—*E. E. M.*

There are too many nursing homes already, and we certainly cannot advise you to start one in any locality, especially as neither you nor your sister is a trained nurse.

*Monthly Nursing.*

(133) Where would you advise any one going in for monthly nursing to go for training? Can you tell me how cases are got after training?—*Bedford.*

See "How to Become a Nurse. The Nursing Profession: How and Where to Train." After training cases are obtained (*a*) by joining an institution; (*b*) through the introduction and recommendation of medical men; (*c*) privately, or through advertisements.

*Fever Training.*

(134) Will you kindly tell me if there is a hospital where I can obtain a free training as fever nurse?—*D. W.*

Fever training is usually obtained free. For list of hospitals see: "How to Become a Nurse. The Nursing Profession: How and Where to train."

*Peace Pillow.*

(135) Please inform me where the pillows can be obtained which are either electrified or else made of something to produce sleep? I have seen them advertised, but cannot remember where.—*Nurse M.*

From the Peace Pillow Company, 17 Manchester Avenue, E.C.

**Handbooks for Nurses.**

| | Post Free. |
|---|---|
| "A Handbook for Nurses." (Dr. J. K. Watson.) ... | 5s. 4d. |
| "Nurses' Pronouncing Dictionary of Medical Terms." ... | 2s. 0d. |
| "Art of Massage." (Creighton Hale.) ... ... ... | 6s. 0d. |
| "Surgical Bandaging and Dressings." (Johnson Smith.) | 2s. 0d. |
| "Hints on Tropical Fevers." (Sister Pollard.) ... ... | 1s. 8d. |

**Of all booksellers or of The Scientific Press, Limited, 28 & 29 Southampton Street, Strand, London, W.C.**

### For Reading to the Sick.

"I AM WITH THEE."

Why should we faint and fear to live alone,
Since all alone, so Heaven has willed, we die.
Nor even the tenderest heart, and next our own,
Knows half the reasons why we smile and sigh?

Each in his hidden sphere of joy or woe
Our hermit spirits dwell, and range apart,
Our eyes see all around in gloom or glow—
Hues of their own fresh borrowed from the heart.

And well it is for us our God should feel
Alone our secret throbbings: so our prayer
May readier spring to Heaven, nor spend its zeal
On cloud-born idols of this lower air.

For if one heart in perfect sympathy
Beat with another, answering love for love,
Weak mortals, all entranced, on earth would lie,
Nor listen for those purer strains above.

*Keble.*

Now loneliness is a thing which we must learn to face in our work in the separations of life and in times of quiet. Certainly, whether we like it or not, we must be alone in death as far as this world is concerned. But the good Shepherd says rather learn attachment. It is His promise: "Fear not; I will be with thee." It is our confidence. "I will fear no evil; for Thou art with me." Nay, more; it is our joy. "Who shall separate us from the love of Christ?" And is not this the true answer to our fears—How can I go to meet that shadow? How will my faith stand its cold embrace? How shall I ever believe in the bright promise of a land beyond, when all is dark? Let us ask rather—Is He with me now? Have I learned to find Him in the quiet hours of the day? Have I found His presence in desolating sorrow? Have I felt His hand in darkness and doubt? Have I found Him near me in prayer and Eucharist? If so, I need not look forward. He is leading me on, step by step, and day by day. He is habituating me, little by little, to the withdrawal of the light, and to utter trust in Him. "Sufficient unto the day is the evil thereof." There is grace given me for the new day's work; there is grace given me under this desolating sorrow. There is grace given me to live well; when I need it, there will be grace given me to die well. "For Thou art with me." Now is the time to make firm that companionship. To be still, and know that He is God. To find the guiding Hand in all its strength and security, amid the death and life of each day's hopes and fears. And then, when we enter the shadow, still it will be "with God onwards."—*Canon Newbolt.*

Thou know'st our bitterness—our joys are Thine;
No stranger Thou to all our wanderings wild:
Nor could we bear to think how every line
Of us, Thy darkened likeness and defiled,

Stands in full sunshine of Thy piercing eye,
But that Thou call'st us Brethren: sweet repose
Is in that word! the Lord Who dwells on high
Knows all, yet loves us better than He knows.

*Keble.*

## Figures and Tables

**Fig. 2. f1:**